# White-matter functional topology: a neuromarker for classification and prediction in unmedicated depression

**DOI:** 10.1038/s41398-020-01053-4

**Published:** 2020-10-30

**Authors:** Jiao Li, Heng Chen, Feiyang Fan, Jiang Qiu, Lian Du, Jinming Xiao, Xujun Duan, Huafu Chen, Wei Liao

**Affiliations:** 1 The Clinical Hospital of Chengdu Brain Science Institute, School of Life Science and Technology, University of Electronic Science and Technology of China, Chengdu, 610054 People’s Republic of China; 2 MOE Key Lab for Neuroinformation, High-Field Magnetic Resonance Brain Imaging Key Laboratory of Sichuan Province, University of Electronic Science and Technology of China, Chengdu, 610054 People’s Republic of China; 3 School of Medicine, Guizhou University, Guiyang, 550025 People’s Republic of China; 4 School of Psychology, Southwest University, Chongqing, 400715 People’s Republic of China; 5 Department of PsyCiatry, The First Affiliated Hospital of Chongqing Medical University, Chongqing, 400016 People’s Republic of China

**Keywords:** Neuroscience, Predictive markers

## Abstract

Aberrant topological organization of brain connectomes underlies pathological mechanisms in major depressive disorder (MDD). However, accumulating evidence has only focused on functional organization in brain gray-matter, ignoring functional information in white-matter (WM) that has been confirmed to have reliable and stable topological organizations. The present study aimed to characterize the functional pattern disruptions of MDD from a new perspective—WM functional connectome topological organization. A case-control, cross-sectional resting-state functional magnetic resonance imaging study was conducted on both discovery [91 unmedicated MDD patients, and 225 healthy controls (HCs)], and replication samples (34 unmedicated MDD patients, and 25 HCs). The WM functional networks were constructed in 128 anatomical regions, and their global topological properties (e.g., small-worldness) were analyzed using graph theory-based approaches. At the system-level, ubiquitous small-worldness architecture and local information-processing capacity were detectable in unmedicated MDD patients but were less salient than in HCs, implying a shift toward randomization in MDD WM functional connectomes. Consistent results were replicated in an independent sample. For clinical applications, small-world topology of WM functional connectome showed a predictive effect on disease severity (Hamilton Depression Rating Scale) in discovery sample (*r* = 0.34, *p* = 0.001). Furthermore, the topologically-based classification model could be generalized to discriminate MDD patients from HCs in replication sample (accuracy, 76%; sensitivity, 74%; specificity, 80%). Our results highlight a reproducible topologically shifted WM functional connectome structure and provide possible clinical applications involving an optimal small-world topology as a potential neuromarker for the classification and prediction of MDD patients.

## Introduction

Human brain connectomics parsimoniously balance the local specialization and global integration to embed a small-world topology^1,2^. This optimal architecture ubiquitously persists in both intact and diseased human functional and anatomical connectomes^3–6^. A functional connectome is generally explored in brain gray-matter (GM) using resting-state functional magnetic resonance imaging (fMRI). Anatomical connectomes, based on bundles of axons within white-matter (WM), are normally characterized using diffusion-tensor imaging (DTI). Although DTI can characterize the detailed infrastructure of WM, it fails to uncover brain dynamics within WM or report possible function-activity states in WM.

Newly discovered evidence has revealed that brain WM also contain neural signal dynamics responding to task-induced brain activation, as well as to intrinsic brain activity^7–9^. Resting-state blood-oxygen-level-dependent (BOLD) fMRI signals in WM can be organized into WM anatomical tracts and also strongly correlate with that in GM^9–11^. Beyond brain activity within WM, our previous network-based works indicated that WM functional connectomes exhibited a reliable and stable small-world topology^12^, and further offered a novel applicable neuromarker of general fluid intelligence^13^. The detectable functional organizations of WM were disrupted in various psychiatric and neurological disorders^14–17^. These disturbances of WM functional networks might provide additional functional information for advancing our understanding of neuropsychopathology of brain diseases.

Depression has increasingly been postulated as an alteration of whole-brain connectome organization, which could serve as a specific diagnostic neuromarker and therapeutic evaluation tool^18–20^. These dysfunctional network organizations suggest that patients with major depressive disorder (MDD) may involve an abnormal capacity of the overall information segregation or integrity in the brain’s connectivity network^18,21^. Leveraging graph-analysis, nontrivial topological properties, involving global (e.g., small-worldness, and modular structure) and nodal properties (e.g., efficiency of highly connected hubs) are disrupted during depression^22,23^. At a functional connectome level within the GM, first-episode, drug-naive depressive patients showed altered global properties (i.e., decreased path lengths and higher global efficiency), indicating a shift toward randomization in brain connectomes^21^, which are with the capacity of increased integration and/or decreased segregation^24^. However, an opposite pattern and no significant depression-associated differences also existed in these global measures^25–27^. In addition, at the anatomical connectomes within WM scales, depressive patients also exhibited mixed findings for global network integrity^28–30^. On the basis of various divergent brain connectome findings, several theories have been proposed to explain MDD at the sample heterogeneity level and brain network definition level^20,31^. On a brain network level, it was crucial to firstly elucidate the role of WM functional connectomes because a hypothesis of aberrant WM functional connectomes might provide an additional functional neuromarker for depression “beyond” GM functional and WM anatomical connectomes.

This study aimed to comprehensively investigate the topological organization of WM functional connectomes in unmedicated MDD patients. As noted, small-world topology is an optimized model used to characterize the brain connectome, considering two fundamental organizational principles in the brain: functional segregated and integrated information processing^21,32,33^. Hence, the present study examined whether the small-world topology of WM functional connectomes was a possible unmedicated MDD-related biomarker. This hypothesis was tested in a large discovery sample of unmedicated MDD patients by: (i) utilizing a graph-based connectome approach to characterize topological properties of WM functional connectomes; (ii) quantifying alterations of small-world topology in MDD patients; and (iii) exploring clinical applications including depression severity prediction and disease classification based on small-world topology. Furthermore, the replicated alterations of small-world topology were conducted using a completely independent sample of MDD patients.

## Materials and methods

### Participants

Two independent samples (called discovery and replication samples) of unmedicated MDD patients were recruited. Discovery samples were conducted by Southwest University, China. All MDD patients were recruited from the Psychiatric Department of the First Affiliated Hospital of Chongqing Medical University and diagnosed using the Structural Clinical Interview for Diagnostic and Statistical Manual of Mental Disorders, by experienced psychiatric physicians. All MDD patients underwent assessment for depression severity by using the 17-item Hamilton Depression Rating Scale (HAMD). The discovery samples included 103 unmedicated MDD patients and 252 age- and sex-matched healthy controls (HCs). Briefly, MDD patients were excluded if they: (i) were ≤18 years of age or ≥65 years of age; (ii) had HAMD scores below 8; (iii) had major neurological or other psychiatric disorders; and (iv) had magnetic resonance imaging (MRI) abnormalities, or had any metal or electronic implants. With advertisements and posters, demographically matched HCs were recruited from college and local community using the following criteria: (i) no mood disorder or neurological disorders, (ii) no history of psychiatric illness among their first-degree relatives, and (iii) no history of substance or alcohol dependence. This study was approved by the Ethics Committee of Southwest University and First Affiliated Hospital of Chongqing Medical University. Written informed consent was obtained from all subjects.

Replication samples included 38 unmedicated MDD patients and 30 age- and sex-matched HCs (recruited by advertisements and posters). The methods and results relative to the replication samples are reported in the Supplementary Materials.

### Data acquisition

All participants in the discovery sample underwent both structural and functional image scanning using a Siemens Trio 3.0 T MRI scanner (Siemens, Malvern, PA, USA) at Southwest University, Chongqing, China. The structural images were acquired from a high resolution, T1-weighted magnetization-prepared rapid gradient echo sequence (repetition time = 1900 ms, echo time = 2.52 ms, inversion time = 900 ms, flip angle = 9°, field of view = 256 × 256 mm^2^, matrix = 256 × 256, voxel size = 1 × 1 × 1 mm^3^, and slices = 176). The resting-state fMRI images were obtained using a single-shot, gradient-recalled echo planar imaging sequence (repetition time = 2000 ms, echo time = 30 ms, flip angle = 90°, field of view = 220 × 220 mm^2^, matrix = 64 × 64, voxel size = 3.4 × 3.4 × 3 mm^3^, and slices = 32). For each subject, a total of 242 volumes (484 s) were acquired. All participants were instructed to simply rest with their eyes closed. The data acquisition of the replication sample is detailed in the Supplementary Materials.

### Data preprocessing

All images were preprocessed using DPARSF (v4.3, www.restfrmi.net) and SPM12 toolkits (www.fil.ion.ucl.ac.uk/spm/software/spm12), as in our previous studies^8,12^. Structural images were co-registered with functional images, and segmented into GM, WM, and cerebrospinal fluid (CSF) using a diffeomorphic nonlinear registration algorithm (DARTEL)^34^ in SPM12. Functional images were preprocessed using a workflow^8,11,12,35^ that is described in Supplementary Materials. To create a group-level WM mask, voxels identified as WM across 80% of all subjects were then included^11,12^. To eliminate the impact of deep brain structures, the Harvard-Oxford Atlas (25% probability) was used to remove subcortical nuclei (i.e., bilateral thalamus, putamen, caudate, pallidum, and nucleus accumbens) from the group-level WM mask. Quality control procedure was presented in Supplementary Fig. S1, and described in Supplementary Materials. Finally, 91 unmedicated MDD patients and 225 HCs from the discovery sample, and 34 unmedicated MDD patients and 25 HCs from the replication sample were included in subsequent analyses.

### Construction of WM functional connectomes

**Fig. 1:**
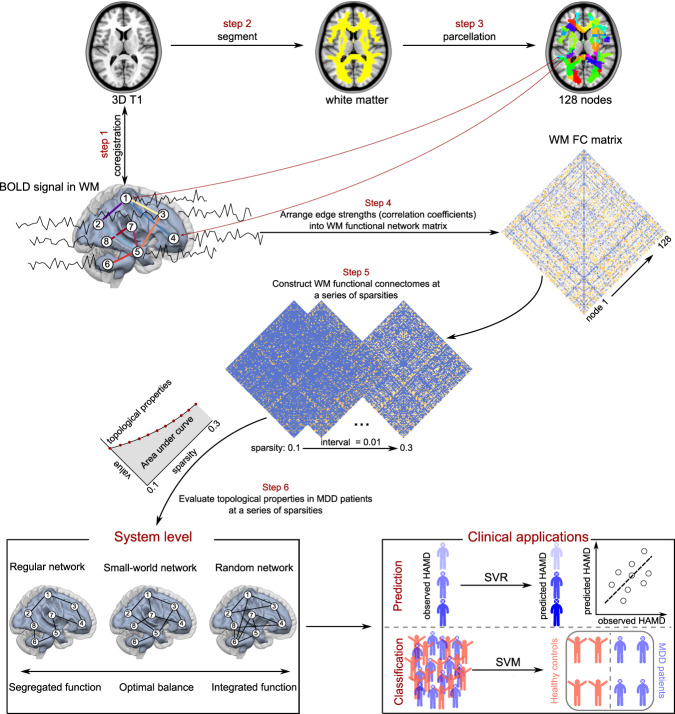
Schematic diagram of the study design. Step 1: Structural images were co-registered with preprocessed functional images. Step 2: The co-registered structural image was segmented into WM, GM, and CSF. Step 3: The group-level WM mask was then randomly separated into 128 anatomical nodes with an approximately identical size. Step 4: BOLD-fMRI signals in WM were then obtained and used to compute FC matrices between each pair of nodes using Pearson’s correlation. Step 5: WM functional connectomes were constructed across a series of sparsity from 0.1–0.3 (interval = 0.01). Step 6: The AUC values of topological properties (i.e., small-world topology and nodal topological properties) were then evaluated across a series of sparsity. Finally, the small-world topology was used as a feature to predict depressive severity and to distinguish the patients from HCs. Abbreviation: AUC, area under curve; BOLD-fMRI, blood-oxygen-level-dependent functional magnetic resonance imaging; CSF, cerebrospinal fluid; FC, functional connectivity; GM, gray matter; HC, healthy control; MDD, major depressive disorder; WM, white matter; SVM, support vector machine; SVR, support vector regression.

To define nodes in WM functional connectomes^12,13,15^, the group-level WM mask was randomly subdivided into *N* (here, *N* = 128) contiguous anatomical regions while constraining the size of nodes as uniformly as possible using a region-growing method^36^. As previously described by Zalesky et al.^36^, *N* seed voxels in WM are randomly chosen, each of which corresponds to the first voxel to be classified as belonging to each of the *N* nodes. All other voxels in WM remain unlabeled. The strategy is to incrementally ‘grow’ each node voxel-by-voxel until every WM voxel has been assigned to exactly one node. At each iteration of the growth phase, a new voxel is assigned to the node with the smallest volume^36^. Each subject’s correlation matrix (128 × 128) was constructed by Pearson’s correlation coefficient between averaged time series within each paired node. Subsequently, Fisher *r* to *Z* transformation was applied to the correlation matrices. Topological properties were evaluated based on the weighted WM functional connectomes. A schematic of the analysis is shown in Fig. 1.

### Topological properties of WM functional connectomes

For WM functional connectomes at each sparsity threshold, the global topological properties were calculated. A small-worldness architecture supported integrated and segregated information processing. Hence, the global topological properties included small-world topology involving the normalized clustering coefficient (*γ*), normalized shortest path length (*λ*), and small-worldness (*σ*) (for a recent review on the interpretations of these network topological properties)^18,33^. These topological properties are known to be interrelated and each provides a different viewpoint from which to discern major features of the large-scale architecture^37^. We then calculated the area under the curve (AUC) of each topological property across the range of sparsities from 0.1–0.3 (interval = 0.01) (Supplementary Fig. S2). Sparse thresholds were determined based on WM FC matrices across all participants from the both discovery and replication samples (Supplementary Materials). The AUC provides a summarized scalar for topological properties of WM functional connectome independent of single threshold selection and was sensitive in detecting abnormalities of topological properties in brain disorder^21^. The global topological properties of WM functional connectomes were computed using Gretna software (v2.0, www.nitrc.org/projects/gretna). The mathematical definitions of these topological properties are listed in the Supplementary Materials.

### Statistical analysis

#### Demographic and clinical characteristics

Demographic and clinical characteristics were evaluated between MDD patients and HCs. Differences in age and education were analyzed using nonparametric Mann–Whitney *U* test (i.e., values did not follow a Gaussian distribution) or the two-sample *t*-tests (i.e., values followed a Gaussian distribution). The *χ*^2^ test was used for sex comparisons.

#### Global topological property comparisons

Differences in AUC values of global topological properties (including *γ*, *λ*, and *σ*) between groups were examined using nonparametric Mann–Whitney *U* tests (i.e., values did not follow a Gaussian distribution) or two-sample *t*-tests (i.e., values followed a Gaussian distribution). Age, sex, educational level, and head motion (mean framewise displacement (FD) values) were used as covariates. The significance threshold was set at *p* < 0.05. Bonferroni correction was used for three planned comparisons.

### Relationships between small-world topology and depression severity

With no strong a *priori* predictions, we specifically investigated the relationships between the small-world topology (including AUC values of *γ*, *λ*, and *σ*) and depression severity (HAMD scores) in the unmedicated MDD patients using Pearson’s correlation analysis. The significance threshold was set at *p* < 0.05. Bonferroni correction was used for three planned correlations.

### Prediction model based on small-world topology

To further investigate a potential clinical application for prediction in MDD, we predicted the depression severity in both the discovery and replication samples from small-world topology using a linear support vector regression (SVR) model (LIBSVM toolbox v3.22, https://www.csie.ntu.edu.tw/~cjlin/libsvm/)^38^. Internal validation analysis was first performed on the discovery sample. The AUC values of small-world topologies (including *γ*, *λ*, and *σ*) of each patient were used as predictive features. A five-fold cross-validation was used in linear SVR. Five-fold cross-validation represents a good compromise between model bias and variance^39^. Briefly, the MDD patients in discovery sample were randomly divided into five folds. Among them, four folds were used as training sets, and the remaining one fold was selected as the testing set. For each trial of cross-validation, the predictive HAMD score was obtained for each patient in testing set based on the building prediction model^22,23^. Finally, we used the Pearson’s correlation to determine whether predicted HAMD score is correlated with the observed HAMD score in patients with MDD^22,23^. To further externally validate the generalization of the predictive model constructed on the discovery sample, the same procedure was applied to the patients with MDD in the replication sample.

### Classification model based on small-world topology

To investigate another potential clinical application for MDD classification, we distinguished MDD patients from HCs using a support vector machine (SVM) model with sigmoid kernel function (LIBSVM toolbox v3.22, https://www.csie.ntu.edu.tw/~cjlin/libsvm/)^38^. The AUC values of small-world topologies (including *γ*, *λ*, and *σ*) of each patient were used as classification features. Because of the imbalance of the discovery sample numbers (225 HCs vs. 91 MDD patients), an ensemble strategy was used to avoid classification bias^40^. Specifically, in each trial, 80% of MDD patients and HC subjects of equal number were randomly selected to train the SVM classification model. This trial was repeated three times, which covered almost all participants, and an odd value (=3) benefited the subsequent voting procedures. Consequently, three classification models were created and then directly applied to the replication samples, rather than to the discovery sample. Finally, the classification label (1: positive, 0: negative) in replication sample was obtained based on the voting for these three models.

### Network analysis on replication sample

To produce a complete and direct replication, the small-world topologies (including *γ*, *λ*, and *σ*) of WM functional connectomes were re-evaluated in the replication sample. The AUC values of small-world topologies across the same range of sparsity were then compared between MDD patients and HCs using Mann-Whitney *U* tests or two-sample *t*-tests after controlling for confounding factors of age, sex, education, and head motion (mean FD). The significance threshold was set at *p* < 0.05. Bonferroni correction was used for three planned comparisons.

## Results

### Demographic and clinical characteristics

The final analysis included data from 91 unmedicated MDD patients and 225 HCs. No differences in age (*p* = 0.47), sex (*p* = 0.69), and educational level (*p* = 0.06) were found between patients and HCs (Table 1).

**Table 1:** Clinical and demographic characteristics.

Characteristics	Discovery sample			Replication sample		
	Patients (*n* = 91)	HCs (*n* = 225)	*p*-values	Patients (*n* = 34)	HCs (*n* = 25)	*p*-values
Age (years)	36.86 ± 11.51	39.46 ± 15.88	0.47^a^	31.21 ± 8.90	27.56 ± 7.07	0.09^a^
Gender (female/male)	62/29	148/77	0.69^b^	23/11	20/5	0.29^b^
Education (years)	12.51 ± 3.68	13.03 ± 3.92	0.06^a^	12.91 ± 3.21	14.40 ± 2.57	0.06^c^
Duration of illness (months)	29.55 ± 41.52	NA	NA	29.69 ± 38.51	NA	NA
HAMD score	22.30 ± 3.96	NA	NA	22.76 ± 5.95	NA	NA

*HC* healthy controls, *HAMD* 17-item Hamilton Depression Scale, *NA* not available.

Values were mean ± standard deviation (SD).

^a^Mann–Whitney *U* test

^b^Chi-square test.

^c^Two-sample *t*-tests.

### Alterations of small-world topology

**Fig. 2:**
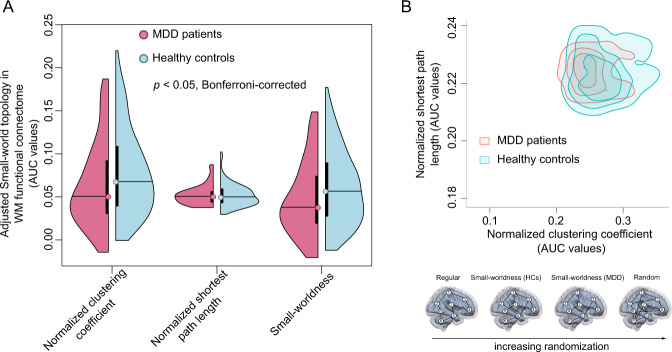
Small-world topology in the patients with MDD and HCs. **a** Between-group comparisons showed the decreased normalized clustering coefficient (Mann–Whitney *U* test, *U* = 8427, *p* = 0.01, Bonferroni corrected), and small-worldness (Mann–Whitney *U* test, *U* = 8434, *p* = 0.01, Bonferroni corrected) in MDD patients compared to HCs. No difference was observed in normalized shortest path length (Mann–Whitney *U* test, *U* = 10,222, *p* = 0.98). The AUC values were adjusted by age, sex, education, and head motion. **b** The patients with MDD exhibited the decreased small-world topology patterns implied as a shift toward randomization in their WM functional connectomes. AUC, area under curve; HC, healthy control; MDD, major depressive disorder.

Small-world topology of WM functional connectomes depends on the choice of sparsity. In the current study, a data specific small-world topology was evaluated at a sparsity range from 0.1–0.3 (internal = 0.01) and were detected in both MDD patients and HCs. However, the patients exhibited a significantly decreased normalized clustering coefficient (*γ*, Mann–Whitney *U* test, *U* = 8434, *p* = 0.01, Bonferroni-corrected), and small-worldness architecture (*σ*, Mann–Whitney *U* test, *U* = 8427, *p* = 0.01, Bonferroni-corrected) compared with HCs (Fig. 2a). No difference was found between patients and HCs in the normalized shortest path length (*λ*, Mann–Whitney *U* test, *U* = 10,222, *p* = 0.98) (Fig. 2a). These results suggested disrupted and segregated information processing in MDD patients (Fig. 2b). To determine possible head motion effects on our results, we performed correlation analyses between mean FD values and small-world topology across participants in the MDD patients and HC groups, respectively. The results showed no statistically significant correlation between small-world topology and head motion (Supplementary Table S1).

### The relationship between small-world topology and depressive severity

**Fig. 3:**
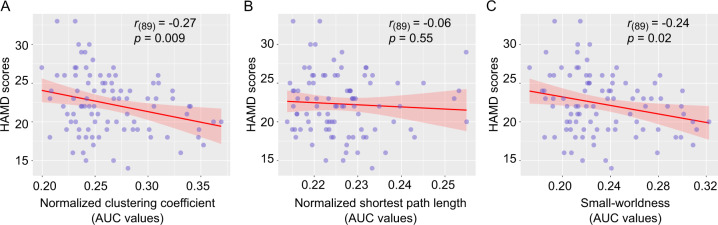
Relationships between small-world topology and depressive severity in MDD patients. **a**–**c** The correlations between normalized clustering coefficients (*r* = –0.27, *p* = 0.009, Bonferroni-corrected), normalized short path length (*r* = –0.06, *p* = 0.55) and small-worldness (*r* = –0.24, *p* = 0.02, uncorrected) and HAMD scores, respectively. AUC, area under curve; HAMD, 17-item Hamilton Depression Scale.

We quantified across-participant relationships between small-world topology (*γ*, *λ* and *σ*) and depression severity by measuring the HAMD. The small-world topology *γ* were negatively correlated with HAMD scores (*r* = –0.27, *p* = 0.009, Bonferroni-corrected) (Fig. 3a), whereas *λ* (*r* = –0.06, *p* = 0.55) (Fig. 3b) and *σ* (*r* = –0.23, *p* = 0.02, uncorrected) (Fig. 3c) did not.

### Prediction and classification results based on small-world topology

**Fig. 4:**
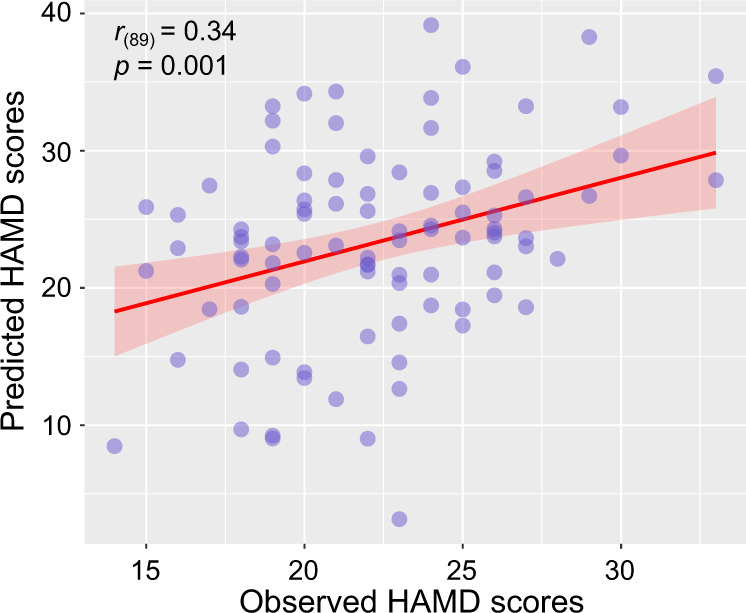
Prediction of HAMD scores based on small-world topology features in MDD patients. The predictive model, based on small-world topology features, could predict HAMD scores in discovery samples using a support vector regression model (*r* = 0.34, *p* = 0.001). HAMD, 17-item Hamilton Depression Scale.

We showed that a predictive model, based on small-world topology features of discovery samples, could be successfully applied to internal validation analyses. We found a significant correlation between observed and predicted HAMD scores in the discovery sample (*r* = 0.34, *p* = 0.001) (Fig. 4). However, this predictive model did not predict HAMD scores on replication samples (*r* = 0.24, *p* = 0.17). We speculated that this generalized prediction tendency might be due to the heterogeneity of scanning parameters and depression severity evaluation procedures.

To further clarify the important role of small-world topology in WM functional connectomes in unmedicated MDD patients, we trained the SVM based on small-world topology in the discovery sample. We then obtained a classification accuracy of 76% (sensitivity = 74% and specificity = 80%) in the replication sample.

### Validation of topological properties in replication samples

We validated alterations of small-world topology in the replication sample. MDD patients in the replication sample also showed decreased global topological properties including *γ* (two-sample *t*-tests, *t* = –2.19, *p* = 0.03, uncorrected) and *σ* (two-sample *t*-tests, *t* = –2.40, *p* = 0.02, uncorrected) compared with HCs, whereas no difference was observed in *λ* (two-sample *t*-test, *t* = 1.41, *p* = 0.16) (Figure S3). Furthermore, we did an auxiliary analysis using 2 (patients and HCs) × 2 (discovery and replication samples) two-way analysis of variance. We did not find statistically significant interaction effect for small-worldness (*p* = 0.06), thus excluding the confounding factor of scanning parameter.

## Discussion

Our findings suggest that integrated and segregated information processing in the human brain are the potential hotspots for aberrant WM functional connectomes in MDD. Aberrant WM functional organizations were examined in both discovery and replication samples, indicating a replicated less integrated function in patients with MDD compared with HCs. Furthermore, the detectable small-world topology predicted the depression severity in MDD patients and distinguished these patients from HCs, highlighting a new index for understanding the pathological mechanisms of MDD.

### Neuromarker of MDD-related topological properties

In the current study, we included MDD patients who were not treated to rule out the heterogeneity of medication^18^. Therefore, our findings suggested that the abnormal small-world topology of WM functional connectomes were unlikely attributable to medication.

The human brain is the most complex network known to man. The rudimentary small-world topology balances functional segregation and specialization, supporting a parsimonious architecture for human brain organization^32^. This small-world topology was replicated in both WM structural and GM functional connectomes of unmedicated MDD patients^28,30^. However, from brain dynamics within WM functional perspective, our previous study elucidated that the WM functional connectome also had reliable and stable small-world topology after controlling for potential confounding factors, such as head motion, parcellation, Euclidean distance, global, and CSF signal regression hemodynamic response function, and thresholding effects^12^. The ubiquitous small-worldness architecture was detectable in unmedicated MDD patients, suggesting an optimal organization of the brain to enable efficient information transfer of distributed processing. The decreased *σ* organization might be attributed to decreased *γ* value, because of the similar pattern of *λ* between unmedicated MDD patients and HCs. A high clustering refers to the ability of densely interconnected groups of brain regions to perform specialized processing procedures related to MDD^24^. The decreased *γ* may reflect disrupted neuronal segregation between interconnected brain regions and is consistent with previous MDD studies based on GM functional or WM structural connectomes^41,42^. In addition, these abnormal patterns of WM functional connectomes were somewhat different from some previous findings regarding WM structure and GM function in depressive patients^18,23,28,30^, such as higher global efficiency of GM functional connectomes in depressed patients, and increased path length of structural connectome in patients with remitted geriatric depression. The heterogenetic results may be caused by brain network definition level. Thus, the identification of potential WM functional features may help us to investigate complementary biomarker for understanding the underpinning mechanism of MDD well.

### Potential clinical applications for MDD

With the evidence pointing to abnormal small-world topology in MDD patients, we performed several exploratory analyses to test whether the small-world topology of WM functional connectomes was strong enough to be considered as potential biomarkers for MDD clinical prediction and classification. For prediction application, the small-world topology of WM functional connectomes could be used as features to predict HAMD scores in the discovery sample. Although the predictive model could not perfectly predict HAMD scores in replication samples, it showed a marginal tendency. This phenomenon might be due to the heterogeneity of scanning parameters and depression severity evaluations and the relatively smaller sample size between the discovery and replication samples. For classification application, the current results distinguished unmedicated MDD patients from HCs using small-world topology of WM functional connectomes. However, MDD patients with the less segregated information processing in WM functional connectome exhibited most depressive severity. The combined two patterns suggested that the identified small-worldness parameters of WM functional connectomes were perhaps indicative of ‘trait’ markers, which were generally associated with clinical status and classification. In addition, the prediction and classification approaches have been proven to successfully differentiate between MDD patients with and without suicidal ideation based on topological properties of GM functional connectomes^23^. Future studies may examine whether applying the small-world topology of the brain connectome facilitates the identification of MDD and further provides early indications for psychiatric treatment.

### Elaborating the possible physiological basis of BOLD fMRI signals in WM

Despite brain connectome studies emerging in depression research, findings from WM functional connectome are still limited. This deficiency may be due to the longstanding controversy about concerning a possible physiological basis in WM. The first concern is that WM contains lower cerebral blood flow and volume compared to GM^7^, resulting in lower BOLD fMRI signals and weaker correlations within WM relative to GM. In addition, BOLD signals are associated with local field action potentials in GM, while not reflecting action potentials in WM^7,43^. However, in comparison to GM, and regardless of large discrepancies with respect to the physiological factors observed between GM and WM, WM maintains a higher ratio of glial cells to neurons^44^, while showing an approximately equal oxygen extraction fraction^45^. Finally, BOLD fMRI signals in WM are always regarded as noise or artifact to be regressed out during resting-state fMRI preprocessing and often are overlooked with application of GM masks during fMRI analyses^11^. However, recent studies have reported existing functional information in WM using resting-state fMRI^7–9,11,12^, suggesting that there are no fundamental barriers or direct sources of evidence against the possibility of detecting WM neural activities using BOLD-fMRI.

Although the functional information was detected in WM, an existing issue is whether WM BOLD-fMRI signals are not interfered by GM neural activities. From the architecture of brain venous systems, the deoxygenated blood from WM is almost independent from cortical GM. In fact, there are two venous systems in normal neuroanatomy: one is the superficial venous system, which drains deoxygenated blood in superficial WM and then via the GM cortex into pial veins; the other is the deep system draining deoxygenated blood in deep WM into subependymal veins. The brain venous architecture is spatially non-overlapping. Deoxygenated blood drainage from the GM cortex to the deep venous system through WM does exist, but the probability of draining is less than 3%^12,35,46^. Therefore, the BOLD-fMRI signals from WM are almost all from WM from brain venous systems. More importantly, we also applied several methods to ensure that WM BOLD-fMRI signals were not affected by strictly GM signals by controlling the boundary between WM and GM (with 90% threshold on the probability map of WM), masking out GM functional images from WM functional preprocessing, and therefore, identifying participants’ voxels only in the WM to create WM mask^8,11,12^. Thus, the abnormal topological properties of WM functional connectomes are in fact due to alterations in WM BOLD-fMRI signals.

### Limitations and future directions

The current study has several limitations. First, the sample size in designing the replication sample was small. This would result in small statistical power of altered small-world topology which did not survive after Bonferroni correction. Future studies should increase the replication samples to validate the current finding. Second, the heterogeneity of scanning parameters and depression severity evaluations are different between the discovery and replication samples. This would contribute to small predictive power of HAMD scores. Another possibility is that the substantial heterogeneity of depression itself^20^ may not be the most compelling goal to predict diagnostic symptoms as a whole. Third, although we have validated the stability and reliability of small-worldness in WM functional connectome using resting-state fMRI^12^, future study will investigate the stability of topological properties across different cognitive task-based fMRI for better exploring the underpinning mechanism in MDD. Finally, considering the several WM structural connectome studies and the first study exploring WM functional connectomes in MDD patients, we did not include WM structural connectomes. Future studies will investigate whether combining the WM functional connectome with WM structural connectome or GM functional connectome can provide complementary biomarkers for understanding the biological mechanisms of MDD patients, such as the identification of neuromarkers for classification and prediction.

## Conclusions

The current study firstly investigated the topological properties of WM functional connectomes in MDD patients with no medication history. We identified and replicated robust decreased small-world topology in two completely independent samples compared to HCs. The clinical applications based on small-world topology of WM functional connectome suggested the potential biomarker of WM functional connectomes on MDD-related early prognosis and diagnosis. Collectively, the replicated effects in WM functional connectomes provide a novel index of small-world topology alterations in unmedicated MDD that can be readily combined with additional neuroimaging modalities, perhaps yielding more sensitive neuromarkers for MDD.

## Supplementary information

Supplemental information for White-matter functional topology: A neuromarker for classification and prediction in unmedicated depression

## Data Availability

The global topological properties were computed using Gretna software v2.0 in https://www.nitrc.org/projects/gretna. The predication and classification model were constructed using LIBSVM toolbox v3.22 in https://www.csie.ntu.edu.tw/~cjlin/libsvm/.
