# Supplementary material for: White-matter functional topology: a neuromarker for classification and prediction in unmedicated depression
Source: Transl Psychiatry. 2020 Oct 30;10:365. doi: 10.1038/s41398-020-01053-4 (PMC7603321; doi:10.1038/s41398-020-01053-4)
Supplement: Supplementary file 1 — Supplemental information for White-matter functional topology: A neuromarker for classification and prediction in unmedicated depression [file 41398_2020_1053_MOESM1_ESM.docx]

**Supporting Materials for**

**White-matter functional topology: A neuromarker for classification and prediction in unmedicated depression**

Running title: A neuromarker of MDD from WM functional topology

**Jiao Li^a,b^, Heng Chen^a, b, c^, Feiyang Fan^a,b^, Jiang Qiu^d^, Lian Du^e^, Jinming Xiao^a,b^, Xujun Duan^a,b^, Huafu Chen^a,b*^, Wei Liao^a,b*^**

^a^ The Clinical Hospital of Chengdu Brain Science Institute, School of Life Science and Technology, University of Electronic Science and Technology of China, Chengdu 610054, P.R. China.

^b^ MOE Key Lab for Neuroinformation, High-Field Magnetic Resonance Brain Imaging Key Laboratory of Sichuan Province, University of Electronic Science and Technology of China, Chengdu 610054, P.R. China.

^c^ School of Medicine, Guizhou University, Guiyang 550025, P.R. China.

^d^ School of Psychology, Southwest University, Chongqing 400715, P.R. China.

^e^ Department of Psychiatry, The First Affiliated Hospital of Chongqing Medical University, Chongqing 400016, P.R. China.

* Corresponding authors:

Huafu Chen or Wei Liao, The Clinical Hospital of Chengdu Brain Science Institute, MOE Key Laboratory for Neuroinformation, University of Electronic Science and Technology of China, Chengdu 610054, P.R. China.

E-mail: chenhf@uestc.edu.cn (H. Chen); weiliao.wl@gmail.com (W. Liao).

**Supplementary Methods and Materials**

**Data acquisition in the replication sample**

All subjects of the replication sample underwent structural and functional imaging scanning using a 3.0 Tesla MRI scanner (GE Medical Systems) at the First Affiliated Hospital of Chongqing Medical University, Chongqing, China. The structural images were acquired from a high-resolution, T1-weighted magnetization-prepared rapid gradient echo sequence (repetition time = 8.348 ms, echo time = 3.272 ms, flip angle = 12°, field of view = 240 × 240 mm^2^, matrix = 256 × 256 and zero filled and interpolated to 512 × 512, voxel size = 0.47 × 0.47 × 1 mm^3^, and slices = 156). The resting-state fMRI images were obtained using a single-shot, gradient-recalled echo planar imaging sequence (repetition time = 2000 ms, echo time = 30 ms, flip angle = 90°, field of view = 240 × 240 mm^2^, matrix = 64 × 64, voxel size = 3.75 × 3.75 × 4 mm^3^, and slices = 33). For each subject, a total of 240 volumes (480 s) were acquired. All subjects were instructed to simply rest with their eyes closed. This study was approved by the Ethics Committee of Southwest University and First Affiliated Hospital of Chongqing Medical University. Written informed consent was obtained from all subjects.

**Functional data preprocessing**

For discovery and replication samples, functional images were preprocessed by following steps: i) slice-timing correction and realignment were performed on the remaining 235 functional images after removing the first seven images (14 s) for the discovery sample, and remaining 235 functional images after removing the first five images (10 s) for the replication sample, respectively. Subjects were excluded if the head motion exceeded 3.0 mm translation or 3.0° rotation. Each subject’s mean framewise-displacement (FD) was also calculated. ii) Nuisance signals, including mean CSF signals, 24 head motion parameters (six rigid-body head motion parameters, one time point before, and the 12 corresponding squared items), and scrubbing parameters (FD > 0.5 mm along with one-forward and two-back neighbors) were regressed out in each subject’s individual space. The mean WM and global signals were not regressed out to avoid eliminating the meaningful neural signals [^1-4^](#_ENREF_1).

To minimize the impact of GM signals on WM signals due to partial volume effects, subsequent preprocessing of functional images was performed only for WM signals [^2^](#_ENREF_2)^,^ [^3^](#_ENREF_3). Therefore, individual masking was generated using a rigorous 90% threshold on the probability map of WM, which was obtained in structural image segmentation. The subjects’ WM functional images were then spatially extracted from WM individual masks using the dot product. iii) WM functional images were then normalized to Montreal Neurological Institute (MNI) space and resampled to 3 × 3 × 3 mm^3^ using the deformation fields, which were generated by the DARTEL template registering to the MNI space. iv) Band-pass filtering (0.01–0.10 Hz) were performed to minimize some drifts and non-neuronal sources to BOLD fMRI signals. v) To avoid generating spurious local spatial correlations between voxels, smoothing was not performed. vi) The scrubbing method was performed to reduce any spurious correlations, because of functional connectivity sensitive to head motions. If the FD of a point exceeded 0.5 mm, the value of the signal at the point, as well as one forward and two back neighbors, were removed. vii) Subjects with at least 80% of the functional images (volumes) were included in subsequent analyses.

**Quality control procedures**

After data preprocessing and WM functional network construction, we conducted a data quality control procedure [^5^](#_ENREF_5). First, three HCs whose who head motion exceeded 3.0 mm of translation or 3.0° of rotation and remaining functional images (volumes) less than 80% of whole scanning timepoints after scrubbing analysis were excluded. The qualities of preprocessed functional images were then visually checked, excluding an additional 12 subjects (seven HCs and five patients) with poor quality functional images (e.g., most of the temporal lobe was uncovered or the distribution of functional correlation coefficients did not follow a normal distribution). Subsequently, 24 subjects (17 HCs and seven patients) were excluded because of the small size of giant connected cluster (see details below details), which meant the terrible WM functional connectome constructed in these subjects. Finally, 91 unmedicated MDD patients and 225 HCs were included in subsequent analyses (Figure S1).

In the replication sample, 38 unmedicated MDD patients and 30 HCs were firstly enrolled in this study. First, four HCs and four MDD patients whose who head motion exceeded 3.0 mm of translation or 3.0° of rotation and remaining volumes were less than 80% of whole scanning timepoints after scrubbing analysis were excluded. The quality of preprocessed functional images was then visually checked, excluding an additional one HC with low quality functional images (e.g., most part of the temporal lobe was uncovered or the distribution of functional correlation coefficients did not follow to a normal distribution). Finally, 34 unmedicated MDD patients and 25 HCs were included in subsequent analyses.

**Threshold selection**

To better perform group comparisons of topological properties, a sparsity threshold strategy was applied to the functional connectivity matrix to control same edge numbers across subjects. Sparsity was defined as the ratio of the existing number of edges divided by the maximum possible number of edges in a given network at a$r_{thr}$. We decreased the *r_thr_* from 1 to 0 (from maximum to minimum) until the existing number of edges satisfied a sparsity threshold. Specifically,

$$0\leq\mathrm{sparsity}\leq1=\frac{\varepsilon_{r_{thr}}}{N(N-1)/2}$$

where $\varepsilon_{r_{\mathrm{thr}}}$ expresses the existing number of edges generated by thresholding at $r_{\mathrm{thr}}$, and $N(N-1)/2$ represents the maximum possible number of edges existing in a given network of N nodes [^3^](#_ENREF_3)^,^ [^6^](#_ENREF_6).

Instead of using a single sparsity threshold, a range of sparsity was used to avoid the influence of threshold on topological property comparisons [^7^](#_ENREF_7). The minimum sparsity was defined as follows: i) the average of all node degree (a node degree was the number of connections to this node) in threshold weighted matrices was greater than 2 × log(*N*) ≈ 9.7 with *N* = 128 here; ii) the minimum sparsity was then reevaluated to ensure that the size of the giant connected cluster (or called largest connected component)was higher than 80% of the total number of nodes in the network (Figure S2). The largest connected component of an undirected graph is a subgraph in which any two nodes are connected to each other by paths, and which is connected to no additional node in the subgraph [^8^](#_ENREF_8)^,^ [^9^](#_ENREF_9)[refs]. The maximum sparsity ensured the lowest significant correlation coefficient (*p* < 0.01) among all WM functional connectivity matrices. This procedure minimized the number of spurious edges [^3^](#_ENREF_3)^,^ [^7^](#_ENREF_7)^,^ [^10^](#_ENREF_10). A range of sparsity (0.1–0.3, interval = 0.01) was generated to evaluate the topological properties of WM functional connectomes for all subjects.

**Global topological properties**

The mathematical definitions of the global topological properties were list as below [^11^](#_ENREF_11):

The global topological properties included small-worldness parameters that were an attractive model to characterize brain functional connectome combining functional segregated and integrated information processing.

*Small-worldness properties:*

Small-worldness properties are related to *C_net_* and *L_net_*.

*Clustering coefficient:*

$$C_{i}=\frac{{\sum_{j,h\in G} (w_{ij}w_{ih}w_{jh})}^{1/3}}{K_{i}(K_{i}-1)}$$

where *C_i_* is the weighted clustering coefficient of a node *i*; *w_ij_* is the weight correlation coefficients between node *i* and node *j* in the network *G*, and *K_i_* is the strength of node *i*. The network clustering coefficient was computed by the average of *C_i_* across all nodes in the network. The equation is:

$$C_{net}=\frac{1}{N}\sum_{i\in G} C_{i}$$

*Shortest path length:*

$$L_{net}=\frac{1}{1/(N(N-1))\sum_{i=1}^{N} \sum_{i\neq j}^{N} 1/L_{ij}}$$

where *N* is the number of nodes; *L_ij_* is the shortest path length between nodes i and *j*.

To examine small-worldness properties, WM functional connectomes were generally compared to random networks. A small-world network has similar path length but higher clustering than a random network [^12^](#_ENREF_12), that is *γ* = *C_net_*/*C_random_* > 1, *λ* = *L_net_*/*L_random_* ≈ 1. These two conditions can also be summarized into a scalar quantitative measurement, the small-worldness, *σ* = *γ*/*λ*, which is typically > 1 [^9^](#_ENREF_9)^,^ [^13^](#_ENREF_13). For each individual WM functional network, a set of 100 comparable random networks with similar degree sequence and symmetric adjacency matrix were formed. The *C_random_* and *L_random_* were defined as the average weighted clustering coefficient and weighted path length.

**Supplementary Tables**

**Table S1.** Correlation coefficients between mean frame-displacement (FD) and small-world topology in WM functional connectome across subjects.

| Small-world topology  *p* values | Normalized clustering coefficient | Normalized path length | Small-worldness |
| --- | --- | --- | --- |
| MDD patients | 0.68 | 0.78 | 0.56 |
| HCs | 0.81 | 0.53 | 0.93 |

*Note*. Abbreviations: WM, white-matter; MDD, major depression disorders; HCs, healthy controls.

**Supplementary Figures**

**Figure S1**

**
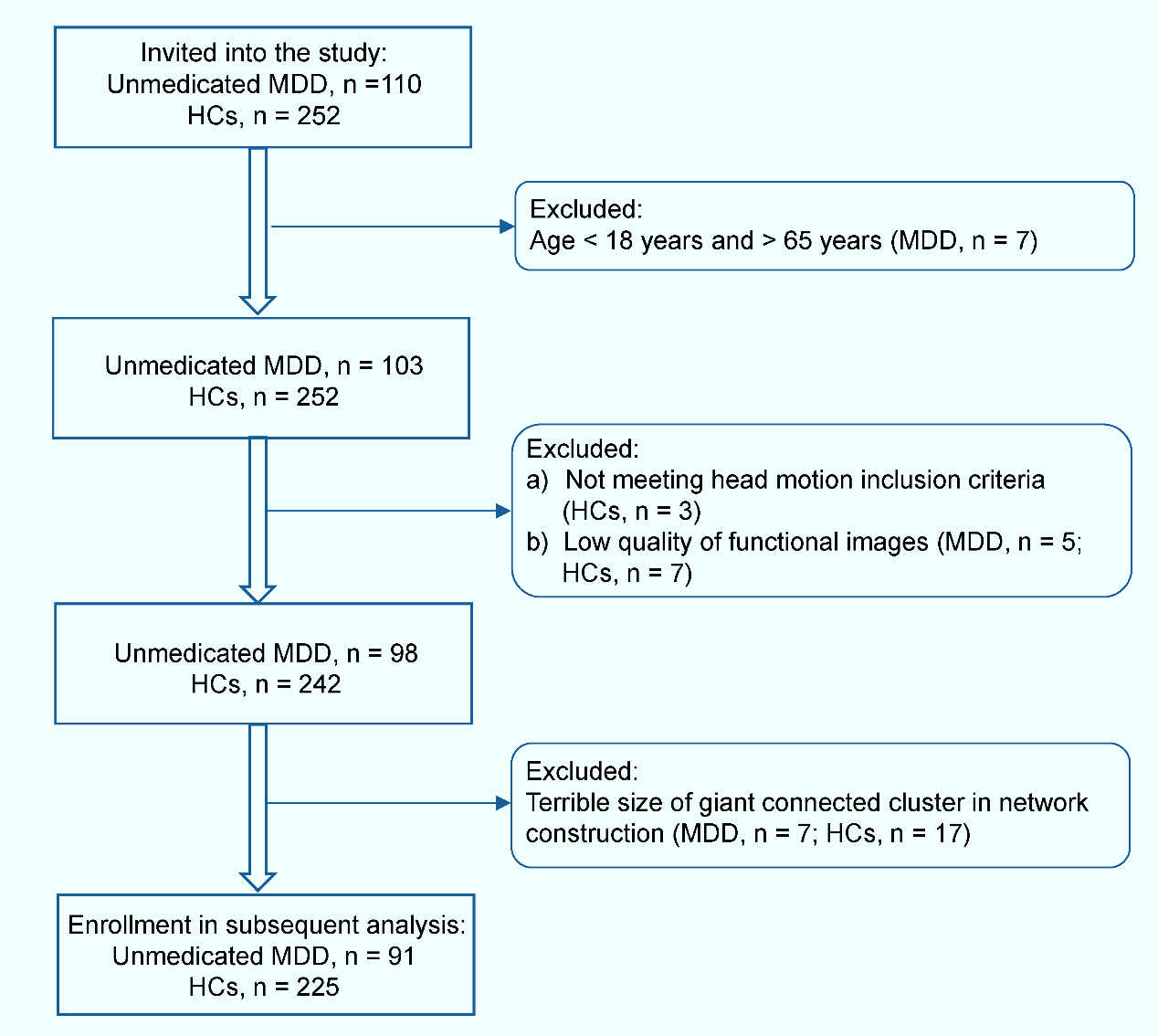
**

**Figure S1.** Flowchart for subject selection.

**Figure S2**

**
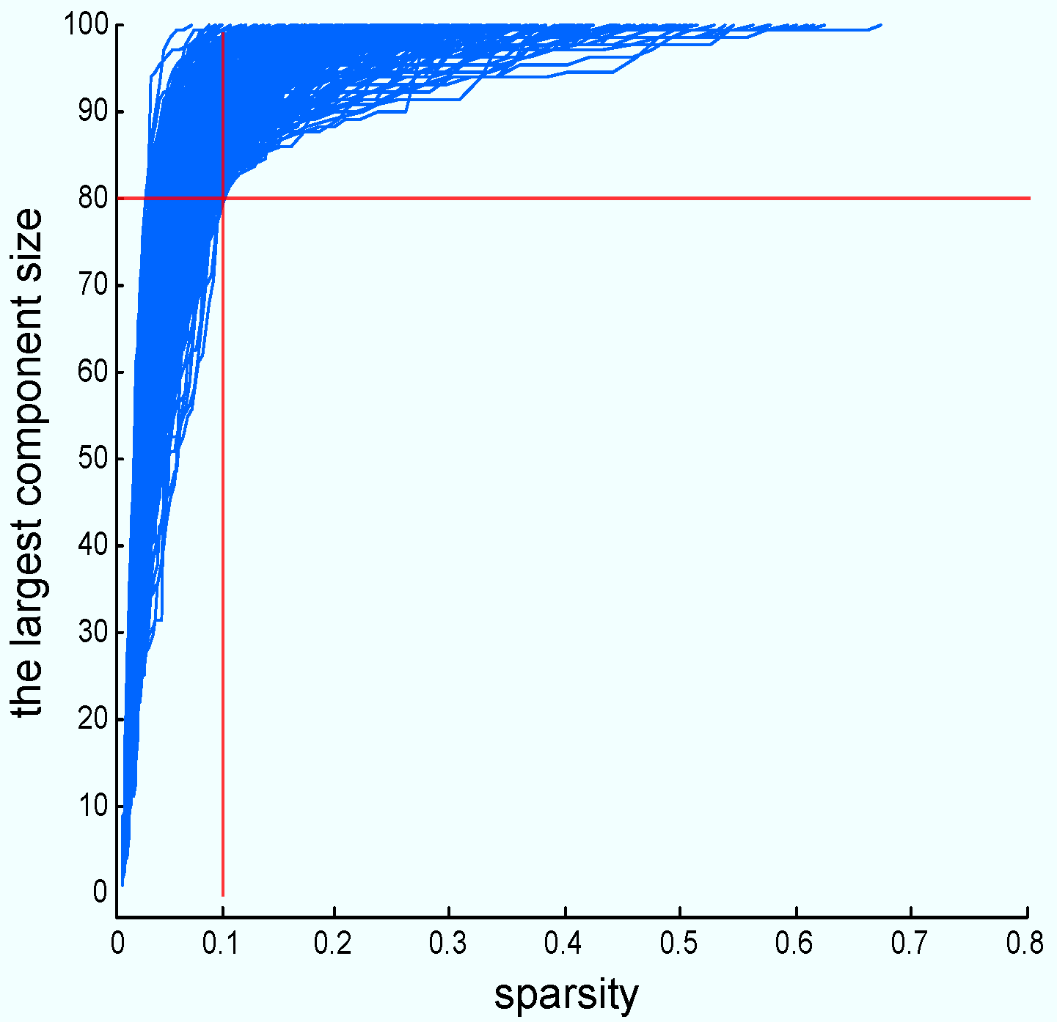
**

**Figure S2.** The size of the largest connected component of WM functional connectomes. The graph showed the largest component size of individual networks as a function of minimum sparsity threshold. When the largest component was set at 80% across subjects, we found the minimum sparsity threshold was 0.10.

**Figure S3**


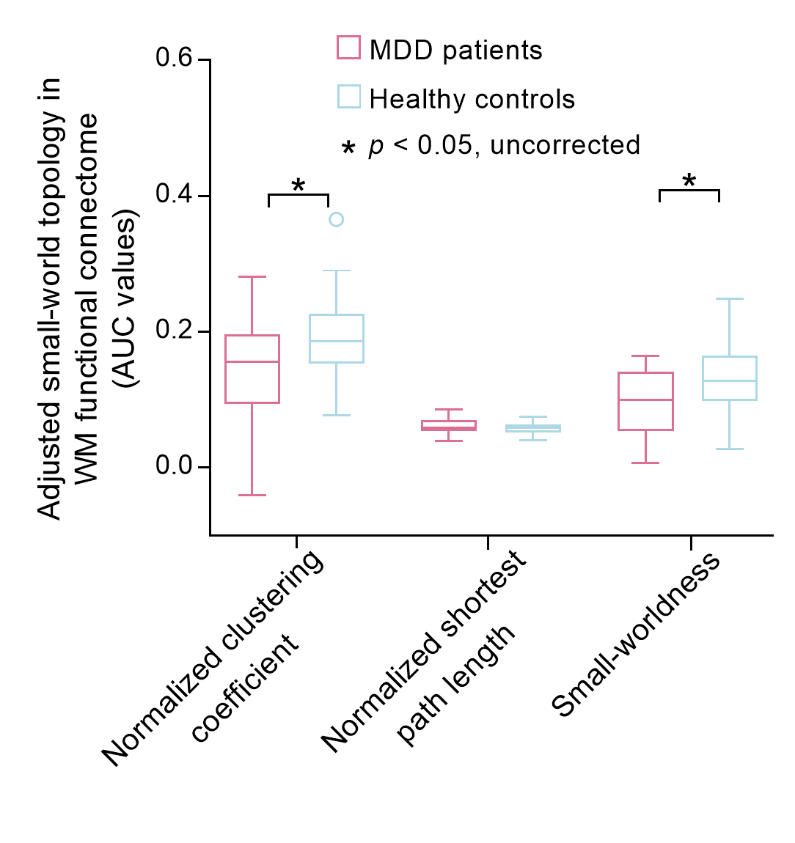


**Figure S3.** **Validation findings of small-world topology in the replication sample.** The patients with unmedicated MDD showed reduced normalized clustering coefficients and small-worldness (*p* < 0.05, uncorrected). The AUC values were adjusted by age, sex, education, and head motion. No difference was observed in normalized shortest path length. Abbreviations: MDD, major depressive disorder.

**References**

1 Huang, Y. et al. Voxel-wise detection of functional networks in white matter. *Neuroimage* **183**, 544-552 (2018).

2 Ji, G. J. et al. Low-frequency blood oxygen level-dependent fluctuations in the brain white matter: more than just noise. *Sci Bull* **62**, 656-657 (2017).

3 Li, J. et al. Exploring the functional connectome in white matter. *Hum Brain Mapp* **40**, 4331-4344 (2019).

4 Peer, M. et al. Evidence for functional networks within the human brain's white matter. *J Neurosci* **37**, 6394-6407 (2017).

5 Liao, W. et al. Altered relationship between thickness and intrinsic activity amplitude in generalized tonic-clonic seizures. *Sci Bull* **61**, 1865-1875 (2016).

6 Bullmore, E. T. & Bassett, D. S. Brain graphs: graphical models of the human brain connectome. *Annu Rev Clin Psychol* **7**, 113-140 (2011).

7 Liao, W. et al. Static and dynamic connectomics differentiate between depressed patients with and without suicidal ideation. *Hum Brain Mapp* **39**, 4105-4118 (2018).

8 Achard, S. & Bullmore, E. Efficiency and cost of economical brain functional networks. *PLoS Comput Biol* **3**, e17 (2007).

9 Achard, S. et al. A resilient, low-frequency, small-world human brain functional network with highly connected association cortical hubs. *J Neurosci* **26**, 63-72 (2006).

10 Ji, G. J. et al. Decreased network efficiency in benign epilepsy with centrotemporal spikes. *Radiology* **283**, 186-194 (2017).

11 Rubinov, M. & Sporns, O. Complex network measures of brain connectivity: Uses and interpretations. *Neuroimage* **52**, 1059-1069 (2010).

12 Watts, D. J. & Strogatz, S. H. Collective dynamics of 'small-world' networks. *Nature* **393**, 440-442 (1998).

13 Humphries, M. D., Gurney, K. & Prescott, T. J. The brainstem reticular formation is a small-world, not scale-free, network. *Proc Biol Sci* **273**, 503-511 (2006).
